# Protective Effects of Melatonin on Retinal Inflammation and Oxidative Stress in Experimental Diabetic Retinopathy

**DOI:** 10.1155/2016/3528274

**Published:** 2016-04-06

**Authors:** Tingting Jiang, Qing Chang, Jiyang Cai, Jiawen Fan, Xiaozhe Zhang, Gezhi Xu

**Affiliations:** ^1^Department of Ophthalmology, Eye and ENT Hospital, Fudan University, Shanghai 200031, China; ^2^Shanghai Key Laboratory of Visual Impairment and Restoration, Shanghai 200031, China; ^3^Department of Ophthalmology, University of Texas Medical Branch, Galveston, TX 77555, USA

## Abstract

Oxidative stress and inflammation are important pathogenic factors contributing to the etiology of diabetic retinopathy (DR). Melatonin is an endogenous hormone that exhibits a variety of biological effects including antioxidant and anti-inflammatory functions. The goals of this study were to determine whether melatonin could ameliorate retinal injury and to explore the potential mechanisms. Diabetes was induced by a single intraperitoneal (i.p.) injection of STZ (60 mg/kg) in Sprague-Dawley rats. Melatonin (10 mg kg^−1^ daily, i.p.) was administered from the induction of diabetes and continued for up to 12 weeks, after which the animals were sacrificed and retinal samples were collected. The retina of diabetic rats showed depletion of glutathione and downregulation of glutamate cysteine ligase (GCL). Melatonin significantly upregulated GCL by retaining Nrf2 in the nucleus and stimulating Akt phosphorylation. The production of proinflammatory cytokines and proteins, including interleukin 1*β*, TNF-*α*, and inducible nitric oxide synthase (iNOS), was inhibited by melatonin through the NF-*κ*B pathway. At 12 weeks, melatonin prevented the significant decrease in the ERG a- and b-wave amplitudes under the diabetic condition. Our results suggest potent protective functions of melatonin in diabetic retinopathy. In addition to being a direct antioxidant, melatonin can exert receptor-mediated signaling effects to attenuate inflammation and oxidative stress of the retina.

## 1. Introduction

Diabetic retinopathy (DR) is a common, potentially devastating microvascular complication of diabetes. It is a leading cause of acquired blindness among working-age people [1, 2]. Current therapeutic options, such as laser photocoagulation, corticosteroids, antivascular endothelial growth factor agents and vitrectomy, are limited by their considerable side effects. Therefore, developing novel, mechanism-based therapeutic strategies is highly desirable for clinical management of DR patients.

It is now well accepted that oxidative stress and inflammation play important roles in the pathogenesis of DR [3, 4]. Shifting the delicate balance between the production and elimination of oxygen radicals or inflammatory cytokines can result in cellular damage. Oxidative stress also causes increased VEGF production in the retina in early diabetes, which is associated with increased vascular permeability and disruption of the blood-retinal barrier. A strong positive correlation has been found between lipid peroxidation products and VEGF concentration in the vitreous of patients with proliferative diabetic retinopathy [5]. Thus, treatments that limit oxidative and inflammatory effects of diabetes could be highly beneficial to DR patients by reducing or preventing the retinal complications.

Melatonin is synthesized primarily by the pineal gland. It is a highly potent endogenous antioxidant [6–10] and anti-inflammatory [11–13] protein. Previous studies have demonstrated that melatonin could protect against several complications of diabetes such as the pancreatic, renal, liver, neural, and corneal injury [11, 14–17]. The retina is an additional site of melatonin synthesis [18], and its rate of synthesis decreased in the rat model of streptozotocin- (STZ-) induced DR [19]. Moreover, it was recently revealed that melatonin reduced some retinal histopathological changes [20] including apoptosis in diabetic rats. Although a range of protective effects of melatonin have been reported in DR, the underlying molecular mechanisms of its action have not been fully evaluated. We previously reported that melatonin augments cellular antioxidant defenses via the phosphoinositide-3 kinase (PI3K)/Akt-Nrf2 signaling pathway in Müller cells [21]. In our current study, we further explored the* in vivo* effects of melatonin on retina of diabetic rats. Our results showed that the melatonin receptors MT1 and MT2 were upregulated in STZ-induced DR. Melatonin stimulated Akt-Nrf2-mediated antioxidant system while it inhibited NF-*κ*B and its downstream proinflammatory cytokine production. The findings suggest that modulating retinal melatonin and its receptor can be a potential interventional strategy for DR.

## 2. Materials and Methods

### 2.1. Animals

Male Sprague-Dawley (SD) rats at 200 to 250 g of body weight were obtained from the Animal Center of the Chinese Academy of Sciences, Shanghai, China. The rats were housed in temperature- and humidity-controlled animal quarters with a 12 h light/dark cycle and were fed with standard rat chow and had free access to water. All the experiments were conducted in accordance with the Association for Research in Vision and Ophthalmology Statement for the Use of Animals in Ophthalmic and Vision Research.

### 2.2. Experimental Design and Induction of Diabetes

Animals were divided into three groups: control nondiabetic, diabetic, and melatonin- (MT-) treated diabetic rats. Diabetes was induced by a single intraperitoneal (i.p.) injection of STZ (60 mg kg^−1^ body weight; Sigma-Aldrich, St. Louis, MO) freshly prepared in citrate buffer (10 mM, Na citrate; pH 4.5). Rats in the nondiabetic group were injected with the same volume of citrate buffer. Three days later, the development of diabetes was confirmed by measuring glucose levels in blood samples taken from the tail vein. Animals with a glycemia level of 16.7 mmol/L (300 mg/dL) or above were considered diabetic and were included in the experimental group. Over 80% of the diabetic rats survived after 12 weeks. After diabetes induction, melatonin (10 mg kg^−1^ daily) (Sigma-Aldrich, St. Louis, MO) was administered by intraperitoneal injection to the animals in the MT-treated group, for 4, 8, or 12 weeks. Dosage of melatonin for each animal was adjusted for its body weight over the entire period of the study. Animals in the nondiabetic and the diabetic (without MT treatment) groups were injected with the solvent (saline with 5% ethanol) as given to the MT-treated group. Body weight and glycemia were measured twice a week throughout the entire course of the experiments. At corresponding time points, rats in each group were sacrificed and their retinas were removed for histological and biochemical examinations.

### 2.3. Preparation of Nuclear and Cytoplasmic Extracts

Pooled retinas were homogenized with a Dounce mechanical homogenizer in five volumes of Buffer A (10 mM KCl, 1.5 mM MgCl_2_, 4 mM *β*-mercaptoethanol (BME), 0.5 mM phenyl-methylsulfonyl-fluoride (PMSF), 10 *μ*g/mL leupeptin, and 10 mM Hepes, pH 7.5) and protease inhibitors. After 10 min incubation at 4°C, samples were centrifuged at 10,000 g, 4°C for 10 min and the supernatant fractions were collected as the cytosolic cell extracts. The pellets were further resuspended in 5 volumes of Buffer B (0.2 mM EDTA; 4 mM BME; 0.5 mM PMSF; 10 *μ*g/mL leupeptin; 20% glycerol; 20 mM Hepes, pH 7.5; 0.3 M ammonium sulfate, pH 7.9) and shook rigorously for 30 min at 4°C. Nuclear extracts were collected after centrifugation at 10,000 g for 10 min. Protein concentrations of both cytosolic and nuclear fractions were measured with the BCA assay.

### 2.4. Fluorescence Immunohistochemistry

Rats were anesthetized with ketamine and xylazine and tissues were fixed by intracardial perfusion with 4% paraformaldehyde in 0.1 M phosphate buffer (pH 7.4). After perfusion, eyes were removed from the animals, placed in fixative for 4 h at 4°C, and then sequentially transferred to 20% and 30% sucrose in phosphate buffer for 16–20 h at 4°C. Eyes were then embedded and frozen in optimal cutting temperature (OCT) embedding compound (Tissue-Tek, Sakura FineTek, Torrance, CA). Sagittal 10 *μ*m sections were cut on a cryostat microtome and collected on glass slides. Tissue sections were rinsed in phosphate buffered saline PBS (pH 7.4). After methanol permeabilization, samples were blocked with 1% bovine serum albumin for 1 hr and then incubated overnight at 4°C in a humidified chamber with antibodies against melatonin receptors MT1 or MT2, glutamine synthase (Santa Cruz Biotechnology, Santa Cruz, CA), Brn-3 (Abcam, Cambridge, MA), or NF-*κ*B p65 (Thermo Fisher Scientific, Waltham, MA). Afterwards, the tissues were washed three times with PBS/0.1% Tween 20 and then incubated with Alexa Fluor 488- or 555-conjugated secondary antibodies (1 : 1000 dilution, Invitrogen, Grand Island, NY) for 1 h at room temperature. After two further washes in PBS/0.1% Tween 20, the nuclei were further stained with 1 *μ*g/mL of 4′,6-dianidino-2-phenylindole (DAPI) in PBS for 5 min. The slides were mounted with glycerin and images were acquired with a Carl Zeiss LSM 510 confocal microscope.

### 2.5. Real-Time Quantitative RT-PCR

Expression levels of the mRNA transcripts of VEGF, iNOS, IL-1*β*, TNF-*α* were measured by real-time RT-PCR. Retinal total RNA was isolated (Trizol Reagent; Invitrogen), and cDNA was synthesized using M-MLV reverse transcriptase (Promega, Madison, WI) and random hexamer (Applied Biosystems, Foster City, CA). All quantitative PCR reactions were performed on an ABI 7300 system, using the SYBR Green-based detection method (Applied Biosystems). Primer sequences are listed in Table 1. Average threshold cycle (Ct) values were used to determine the relative differences between control and treated groups and were normalized to *β*-actin mRNA in each sample.

### 2.6. Western Blot Analyses

Western blot analyses were performed using specific antibodies. Anti-Nrf2 (H-300) and Anti-IL-1*β* antibodies were purchased from Santa Cruz Biotechnology (Santa Cruz, CA, USA). Anti-iNOS, anti-VEGF, anti-glutamate cysteine ligase (GCL), and anti-*β*-actin antibodies were purchased from Abcam (Cambridge, MA). Other primary antibodies were purchased from Cell Signaling Technology (Beverly, MA, USA). Retinal samples were mechanically lysed in RIPA buffer (50 mM Tris-Cl, pH 7.5, 150 mM NaCl, 1% NP-40, 0.5% DOC, 0.1% SDS) with a cocktail of protease and phosphatase inhibitors (Sigma). Equal amounts of proteins were separated on 10% SDS polyacrylamide gels and transblotted onto polyvinylidene fluoride (PVDF) sheets (Immobilon TM-P, Millipore Corp., Bedford, MA, USA). Western signals were developed using HRP-conjugated secondary antibodies. Images were scanned from X-ray films and the band intensities were quantified with NIH Image J software.

### 2.7. Measurement of Total Glutathione

Total glutathione levels were measured using an assay kit (Beyotime Institute of Biotechnology, Haimen, China) which is based on DTNB (5,59-dithiobis(2-nitrobenzoic acid)), the Ellman reagent, as the assay substrate. Retinas were homogenized, deproteinated, and centrifuged at 10,000 g for 10 min. Supernatants were added to a 96-well plate and assay was then performed according to the manufacturer's instructions. DTNB reacts with reduced glutathione (GSH) to generate 2-nitro-5-thiobenzoic acid (TNB), with peak absorbance at 412 nm. The other reaction product, the GSTNB mixed disulfide, is reduced back to GSH and TNB by glutathione reductase. The amount of total glutathione was calculated based on an external standard curve and normalized to the protein content in each sample.

### 2.8. Electroretinography (ERG)

ERG activity was assessed after 12 weeks of treatment. Briefly, rats after overnight dark adaptation were anesthetized by ketamine and xylazine and their pupils were dilated with 1% tropicamide (Santen Pharmaceutical, Japan). The cornea was topically anesthetized by the 0.4% oxybuprocaine hydrochloride (Santen Pharmaceutical). A gold electrode was placed in contact with the central cornea. Vidisic gel (Bausch & Lomb, USA) was used as a conducting medium for the corneal electrode. A reference electrode was placed hypodermically on central forehead and a grounding electrode was attached to the tail. ERG responses were recorded from both eyes simultaneously with an ESPION Console (Diagnosys LLC, Littleton, MA, USA). The light pulses were delivered with a commercial Ganzfeld stimulator (ESPION ColorDome Handheld Ganzfeld stimulator, Diagnosys LLC). Body temperature of the testing animal was maintained at 37°C with a heating pad. Ten responses to flashes of white light (4 ms, 0.2 Hz) elicited at 60-second intervals from a photic stimulator set at maximum brightness (20 cd·s/m^2^) were amplified and averaged. The stimulus intensities were 0.01 and 20 cd·s/m^2^. Before photopic conditions, there was ten-minute adaption. Responses were stored for analysis after an average of 10–15 individual measurements. The amplitudes of the a-waves were measured from baseline to the troughs of the a-waves, and the amplitudes of the b-wave were determined from the troughs of the a-waves to the peaks of the b-waves. The resultant mean value was used to compute the means of a- and b-wave amplitude for each experimental group.

### 2.9. Statistical Analysis

All experiments were repeated at least three times. Data were analyzed with the SPSS 17.0 software (IBM). Means ± SD were calculated for each group. The significance of differences between two groups was evaluated using Student's *t*-test. For multiple comparisons, one-way ANOVA followed by Tukey's multiple comparison tests was used. Differences were considered statistically significant when the *P* value was less than 0.05.

## 3. Results

### 3.1. Melatonin-Mediated Antioxidative and Anti-Inflammatory Effects in Diabetic Retina

**Figure 1:**
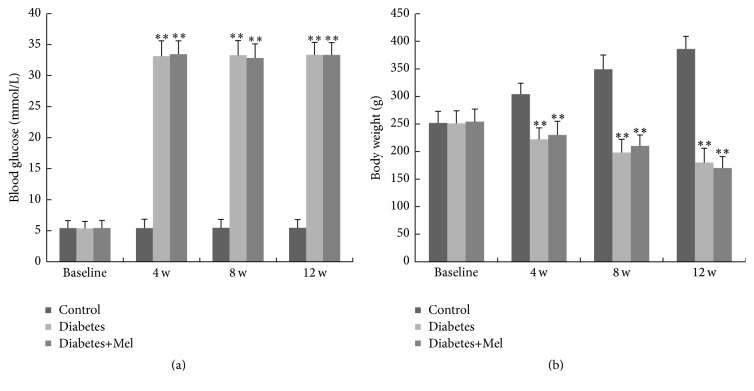
Blood glucose (a) and body weight (b) of rats before (baseline) and after 4, 8, and 12 weeks of treatment. ^*∗∗*^ *P* < 0.01 versus nondiabetic group as determined by one-way ANOVA. Control, nondiabetic group; diabetes, diabetic group; and diabetes+Mel, melatonin-treated diabetic group.

We monitored the changes in body weight and blood glucose in response to STZ and melatonin. The blood glucose levels in diabetic rats with or without melatonin treatment did not differ at the end of the 12-week experiment, and both were significantly elevated than the control nondiabetic group (Figure 1(a)). Animals in all three groups had the same initial body weights (Figure 1(b)). After 12 weeks, control nondiabetic rats had 50% increase in their body weight. In contrast, there was time-dependent decrease in body weight after STZ treatment and melatonin did not affect the loss in body weight in the diabetic animals (Figure 1(b)).

**Figure 2:**
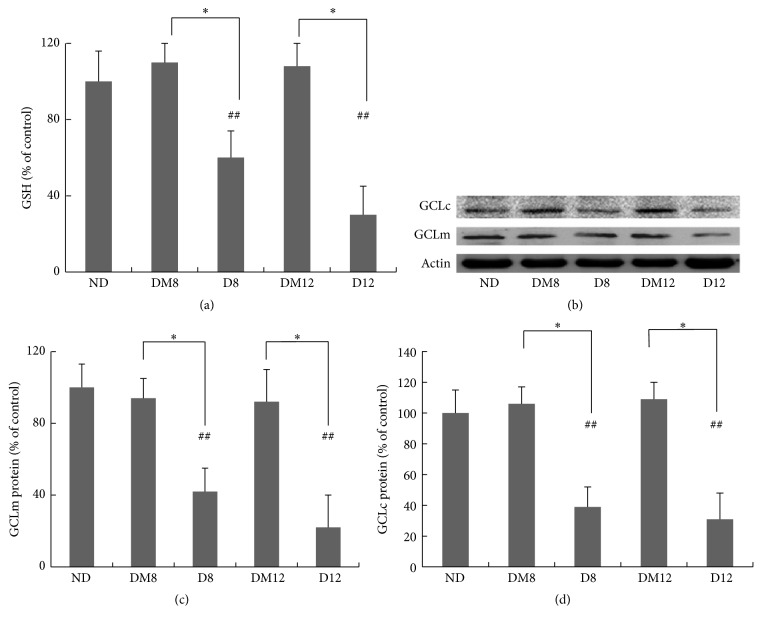
Effect of melatonin on antioxidant GSH and the enzyme GCL expressions in retinas from diabetic rats. (a) Measurement of glutathione contents by the DTNB method. The value from the retinas of the nondiabetic group was considered as 100% (control). (b–d) Western blot of GCL proteins in retinas from the study groups. Quantification data from the Western blot after normalizing to *β*-actin. ^*∗*^ *P* < 0.05 and ^  ##^ *P* < 0.05 when compared with the ND group. ND, nondiabetic group. D8/D12, diabetic group at 8 and 12 weeks; DM8/DM12, melatonin-treated diabetic group at 8 and 12 weeks.

Retinal total glutathione content was measured as an indicator of oxidative stress in the retina. After 8 and 12 weeks of hyperglycemia, STZ-treated rats lost nearly 50% of total glutathione in the retina (Figure 2(a)). Consistently, protein levels of both the modulatory and the catalytic subunits of GCL, the rate-limiting enzyme for GSH synthesis, decreased after 8 weeks (Figure 2). Melatonin treatment prevented the glutathione depletion and GCL downregulation, indicating its antioxidant functions in the diabetic retina.

**Figure 3:**
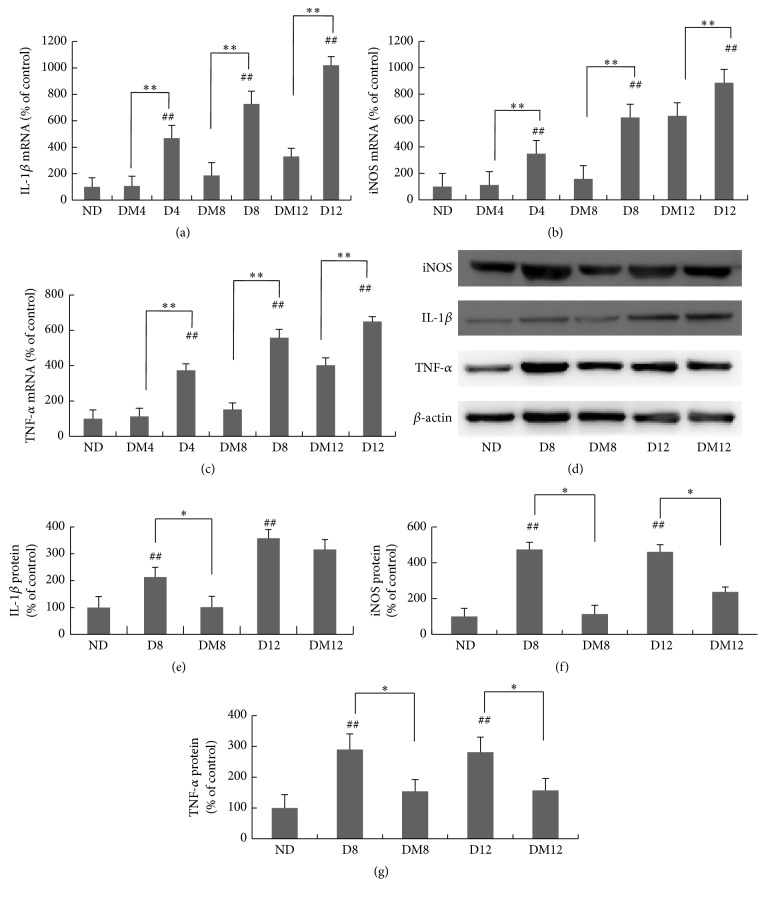
Retinal levels of TNF-*α*, IL-1*β*, and iNOS after melatonin treatment in rats with diabetes. The mRNA levels of IL-1*β* (a), iNOS (b), and TNF-*α* (c) were measured by real-time RT-PCR. (d) Western blot of TNF-*α*, IL-1*β*, and iNOS proteins in retinas from different groups. (e–g) Quantification data from the western blot after normalizing to *β*-actin. ^*∗*^ *P* < 0.05, ^*∗∗*^ *P* < 0.01, and ^##^ *P* < 0.05 when compared with the ND group. ND, nondiabetic group; D4/D8/D12, diabetic group at 4, 8, and 12 weeks; DM4/DM8/DM12, melatonin-treated diabetic group at 4, 8, and 12 weeks.

Inflammatory cytokines are important mediators of diabetic retinopathy. As shown in Figure 3, both the mRNA and the protein levels of TNF-*α*, IL-1*β*, and iNOS were elevated after 4 weeks of diabetes. Melatonin treatment markedly inhibited the expression of those proinflammatory molecules in the retina. Compared to the STZ-alone group, at 8 weeks, melatonin inhibited TNF-*α*, IL-1*β*, and iNOS by 2.65 ± 0.63-, 2.89 ± 0.95-, and 2.94 ± 0.65-fold, respectively (Figures 3(a)–3(c)). The changes at protein levels were also confirmed by immunoblot analysis (Figures 3(d)–3(g)).

### 3.2. Signaling Pathways Regulated by Melatonin in Diabetic Retina

**Figure 4:**
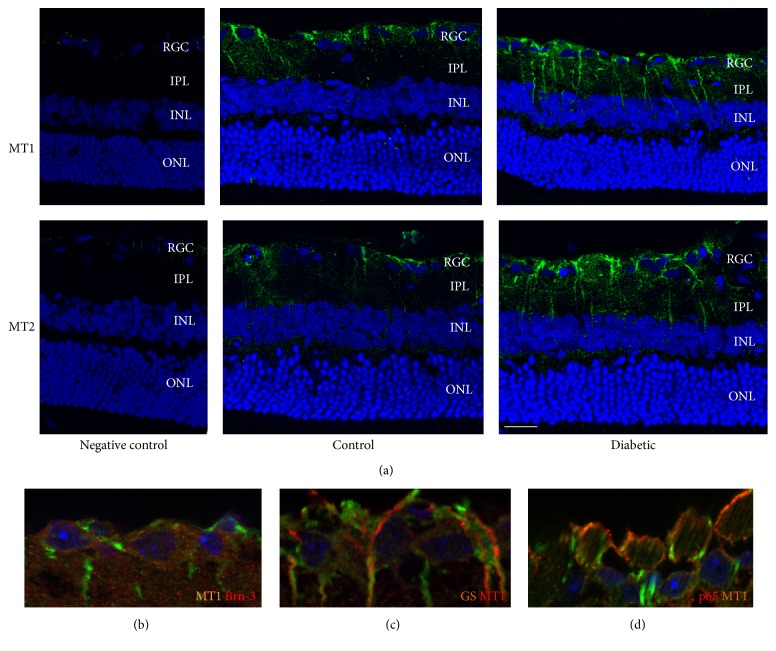
Immunostaining of MT1 and MT2 in nondiabetic and diabetic retinas (a). Immunostaining of glutamine synthase, Brn-3 and NF-*κ*B p65 in nondiabetic retinas (b–d). Nuclei were counterstained with DAPI. In the retinas of nondiabetic rats, specific MT1/MT2 immunolabeling (green) was observed in the ganglion cell layer and in the inner plexiform layer. A further increase in staining intensity of MT1/MT2 occurred at 8 weeks of diabetes. MT1 expression was identified in cells with positive staining of either Brn-3 (b) or glutamine synthetase (c), which are markers of ganglion cells and Muller glial cells, respectively. Immunostaining of the p65 subunit of NF-*κ*B showed colocalization with MT1 expression in the ganglion cell layer (d). Scale bar, 50 *μ*m.

Melatonin has two cell surface receptors, MT1 and MT2. As shown in Figure 4(a), immunocytochemistry using the anti-MT1 or anti-MT2 antibody identified strong and specific immunoreactivity in the ganglion cell layer and inner plexiform layer of the retina. By 8 weeks after diabetes induction, increased expressions of both MT1 and MT2 were observed in the retina of diabetic rats compared with those in the nondiabetic group. MT1 expression was identified in cells with positive staining of either Brn-3 (Figure 4(b)) or glutamine synthetase (Figure 4(c)), which are markers of ganglion cells and Muller glial cells, respectively.

**Figure 5:**
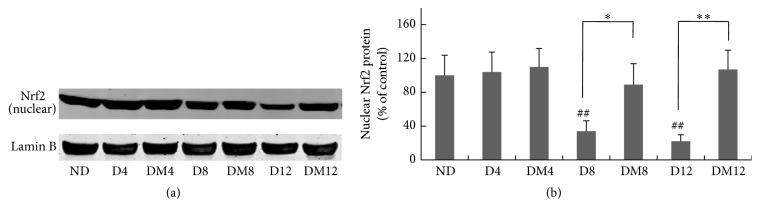
Effect of melatonin on the nuclear Nrf2 expression in retinas from diabetic rats. (a) Western blot of nuclear Nrf2 in retinas from different groups. (b) Quantification data from the western blots after normalizing to Lamin B. ^*∗*^ *P* < 0.05, ^*∗∗*^ *P* < 0.01, and ^##^ *P* < 0.05 when compared with the ND group. ND, nondiabetic group; D4/D8/D12, diabetic group at 4, 8, and 12 weeks; DM4/DM8/DM12, melatonin-treated diabetic group at 4, 8, and 12 weeks.

**Figure 6:**
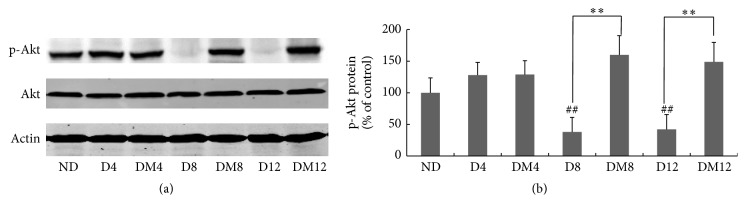
Effect of melatonin on Akt activation in retinas from diabetic rats. (a-b) Akt phosphorylation was measured by western blot analyses. ^*∗∗*^ *P* < 0.01 and ^##^ *P* < 0.05 when compared with the ND group. ND, nondiabetic group; D4/D8/D12, diabetic group at 4, 8, and 12 weeks; DM4/DM8/DM12, melatonin-treated diabetic group at 4, 8, and 12 weeks.

We further explored the* in vivo* effects of melatonin on retinal signaling pathways. Antioxidant responses, including GSH synthesis, are largely controlled by the transcription factor Nrf2. Previous studies reported that the transcriptional activity of Nrf2 decreased in models of diabetic retinopathy. We measured the amount of Nrf2 protein in the nuclear extract of retina. Results (Figure 5) showed that the amount of Nrf2 in the nucleus was markedly decreased in diabetic retinas at 8 and 12 weeks as compared to the control counterparts, and melatonin treatment restored it to control level. In addition to its subcellular location, the transcription activity of Nrf2 is also controlled by the PI3K-Akt pathway. Akt phosphorylation was significantly reduced in retinas of diabetic rats at 8 and 12 weeks but was retained in melatonin-treated animals (Figure 6).

**Figure 7:**
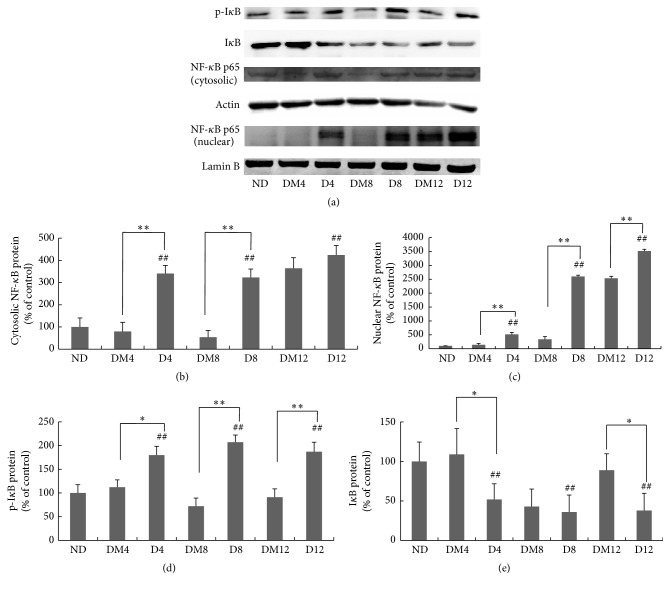
Effect of melatonin on NF-*κ*B expression in retinas from diabetic rats. (a) Western blot of cytosolic NF-*κ*B, nuclear NF-*κ*B, I*κ*B, and phosphorylated I*κ*B proteins in retinas from different groups. (b–e) Quantification data from the western blots after normalizing to *β*-actin. ^*∗*^ *P* < 0.05, ^*∗∗*^ *P* < 0.01, and ^##^ *P* < 0.05 when compared with the ND group. ND, nondiabetic group; D4/D8/D12, diabetic group at 4, 8, and 12 weeks; DM4/DM8/DM12, melatonin-treated diabetic group at 4, 8, and 12 weeks.

Next we explored whether the anti-inflammatory effects of by melatonin can be attributed to the NF-*κ*B pathway. Compared with the values obtained from the nondiabetic group, phosphorylated I*κ*B and the nuclear and cytosolic NF-*κ*B protein were increased in retina of diabetic rats (Figures 7(a)–7(d)). However, melatonin exerted inhibitory effects on the activation of NF-*κ*B and the degradation of I*κ*B (Figure 7(e)). Immunostaining of the p65 subunit of NF-*κ*B showed diffused distribution throughout the inner and outer retina and colocalization with MT1 expression in the ganglion cell layer (Figure 4(d)).

### 3.3. Protective Effects of Melatonin on the Retinal Function in Diabetic Rats

**Figure 8:**
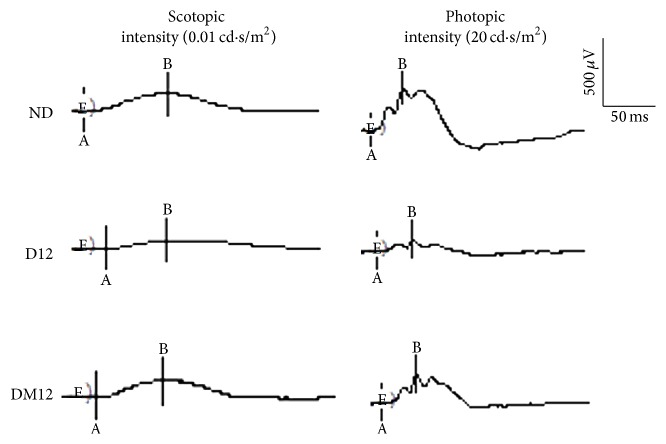
Effect of melatonin on retinal function. Representative ERG traces from all experimental groups. ND, nondiabetic group; D12, diabetic group at 12 weeks; DM12, melatonin-treated diabetic group at weeks.

Scotopic and photopic ERG responses were measured as indicators of retinal functional changes caused by diabetes. At 12 weeks, the average amplitudes of a- and b-wave were significantly reduced in diabetic animals (Figure 8, Table 2). The decreased ERG response was alleviated by melatonin treatment. No statistical differences in the ERG a-wave and b-wave latencies were observed among groups (data not shown).

**Figure 9:**
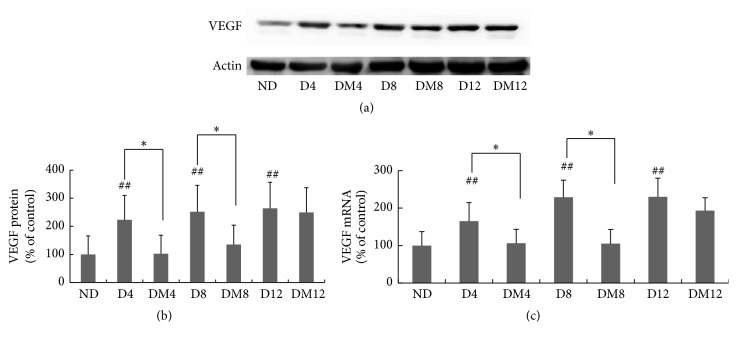
Effects of melatonin on VEGF expression in retinas of diabetic rats. (a) Western blots of VEGF protein in retinas obtained from rats in the three study groups. (b) Quantification data from the western blots after normalizing to *β*-actin. (c) VEGF expression measured by real-time RT-PCR and normalized to *β*-actin mRNA. ^*∗*^ *P* < 0.05, ^*∗∗*^ *P* < 0.01, and ^##^ *P* < 0.05 when compared with the ND group. ND, nondiabetic group; D4/D8/D12, diabetic group at 4, 8, and 12 weeks; DM4/DM8/DM12, melatonin-treated diabetic group at 4, 8, and 12 weeks. As there was no significant difference at all the time points of nondiabetic group, results just showed a random age-matched control group as normal control group.

Dysregulated retinal VEGF production during DR is one of the most devastating responses to oxidative stress [22, 23]. Compared with the nondiabetic group, VEGF mRNA and protein levels were significantly increased in retinas of diabetic rats. However, this upregulation was attenuated with melatonin treatment, significantly at 4 and 8 weeks of treatment (Figure 9).

## 4. Discussion

Melatonin has been found to be a highly effective endogenous antioxidant in various experimental situations [24–26] and it also exerts strong anti-inflammatory effects [27]. Compared to other antioxidant drugs, melatonin has many advantages. It can directly detoxify the highly damaging hydroxyl radical [28]. Furthermore, several of the metabolites that are generated when melatonin inactivates toxic reactants are themselves direct free radical scavengers. Beyond its actions as a direct free radical scavenger, melatonin also stimulates a number of antioxidative enzymes [24]. Melatonin also has been shown to reduce electron leakage at the mitochondrial level, thereby avoiding radical generation [29]. These combined actions of melatonin, along with its low toxicity and its ability to penetrate morphophysiological membranes, make it a ubiquitously acting and highly beneficial antioxidant. Our current study investigated the protective effect of melatonin on diabetic retinopathy in STZ-induced diabetic rats. The STZ model is the most widely accepted animal model for the evaluation of retinal complications in diabetes. Various biochemical and histological alterations closely resemble the initial process of diabetic retinopathy that occurs in humans [30], such as thickening of the basement membrane, microaneurysms, decreased pericyte number, increased vascular permeability, and breakdown of the blood-retinal barrier [31–33]. In addition, reduced electroretinographic (ERG) responses can be detected as early as 2 weeks after the onset of diabetes in rats [34]. We found that melatonin can attenuate diabetic retinal injury by protecting against oxidative and inflammatory stress and preserve the retinal ERG responses (Table 2). The findings are consistent with previously published observations from Salido et al. [26]. In addition, the preventive effect of melatonin on the decrease in the oscillatory potential amplitude and similar functional protection by the delayed treatment with melatonin (started 3 wk after STZ injection) have been reported in their study. Collectively, this further supports the potential application of melatonin in DR.

In the current study, we found that melatonin was unable to ameliorate metabolic abnormalities such as body weight loss and hyperglycemia in diabetic rats (Figure 1). The effects of melatonin on glucose metabolism in a diabetic background are controversial. While we and others only found limited systemic effects [25, 26], some previous studies reported that melatonin could reduce fasting hyperglycemia and improve insulin desensitization [35, 36]. The differences are likely due to the experimental species and models used for the studies, as well as the route of melatonin delivery. Nonetheless, the retina is a site of extrapineal melatonin production and the tissue-specific functions are at least partly mediated through its receptors.

Ocular melatonin receptors have been characterized in the retina of a number of different species, and their distributions have been identified in multiple layers of the retina such as photoreceptors, the inner nuclear layer (INL), and ganglion cell layer (GCL) [37, 38]. In the present study, MT1/MT2 immunohistochemistry showed a high level of labeling, which was primarily restricted to the ganglion cell layer and inner plexiform layer of the nondiabetic retina and was upregulated under diabetic conditions (Figure 4(a)). Mechanisms underlying such change remain to be determined. In our previous study, we demonstrated the expressions of both MT1/MT2 receptors in the Müller cells which are upregulated in hyperglycemic conditions [21]. Immunostaining data (Figure 4(c)) showed the expression of MT1 in Müller cell processes around the ganglion cells. The preventive effect of melatonin on the reactive expression of GFAP in Müller cells, which is widely used as a molecular indicator for retinal stress, has been reported [26]. Thus, we conclude that the increase in MT1 and MT2 levels in diabetic retinas is likely due to Müller gliosis after ganglion cell damage in DR. Another explanation is that it may be a compensatory response as there is a melatonin synthesis reduction in diabetic retinas [19]. MT1 and MT2 participate in many important functions of the retina [39] and the effects of melatonin can be either receptor dependent or receptor independent [40, 41]. In our previous study* in vitro*, we demonstrated that the antioxidative and antiangiogenic effects of melatonin in Müller cells are largely receptor dependent [21]. The role of the two receptors in the protective effect of melatonin in diabetic retinas should be investigated further and additional future studies can be performed to examine the potential neuroprotective roles of those receptor proteins. Given the diversity of cell types that exist in these layers, the cell type-specific distribution of MT1/MT2 receptors in either control or diabetic animals remains unclear and needs to be further investigated.

Previous work from our group demonstrated the cytoprotective effect of melatonin in Müller cells under hyperglycemic conditions [21]. In the present study, we further showed that melatonin can prevent the glutathione depletion in the diabetic retina (Figure 2). GCL is the rate-limiting enzyme of glutathione synthesis [42, 43], and Nrf2 is considered a key transcription factor for the regulation of GCL [44]. When measured for the amount of Nrf2 in nuclear extract of the retina, a significant decrease was observed at 8 and 12 weeks in the diabetic animals (Figure 5), with concomitantly decreased GCL protein level (Figure 2). The results are consistent with a previous study reported by Zhong et al., who showed that although the overall expression of Nrf2 was increased in DR, its nuclear level was decreased [45]. Melatonin treatment prevented the loss of nuclear Nrf2 (Figure 5). The DNA binding activities of Nrf2 at promoter regions of specific downstream genes were not examined in this study and can be further explored in future studies.

Nrf2 is subjected to regulation by a number of upstream signaling pathways, including the PI3K/Akt pathway [46, 47]. In diabetic retina, Akt phosphorylation was markedly reduced (Figure 6) [48, 49]. In our previous* in vitro* study, we showed that melatonin can elevate Nrf2 activity by increasing Akt phosphorylation [21]. Similar effects were observed in the diabetic retina (Figure 6) where melatonin enhanced Akt phosphorylation* in vivo*. Collectively, such findings have shown that melatonin can reduce retinal injury from oxidative stress through the PI3K/Akt-Nrf2 signaling pathway.

Retinal VEGF expression is correlated with diabetic blood-retinal barrier breakdown, increased vascular permeability, and ischemia-related neovascularization [50, 51] and is implicated in the pathogenesis of both background and proliferative diabetic retinopathy [52]. Therefore, therapeutic maneuvers that suppress VEGF overproduction should be able to prevent or attenuate the development or progression of DR. In the present study, treatment with melatonin reduced the elevated VEGF expression considerably in diabetic retinas (Figure 9), which is consistent with previous reports [25, 26]. Such a finding suggests a novel therapeutic effect of melatonin in DR, which may be partly attributed to its antioxidant effects. The protection of melatonin on the functional integrity of the BRB has been reported in the retina of hypoxic rats [53]. In our future study, we will further detect the exact effect of melatonin on the integrity of the BRB by intravenous injection.

Chronic, low-grade subclinical inflammation is responsible for many of the signature vascular lesions of diabetic retinopathy [54]. The levels of proinflammatory cytokines are increased in the retina and vitreous in diabetes [55, 56]. Our study confirmed that TNF-*α*, IL-1*β*, and iNOS expression was markedly upregulated in diabetic retinas. The induction was significantly inhibited by melatonin (Figure 3). Similar results have been reported in the therapeutic effect of melatonin on uveitis [57] and diabetic retinas [26]. NF-*κ*B is a prime molecular target for anti-inflammatory therapy. Its function is based on regulating transcription of multiple inflammatory genes such as TNF-*α*, IL-1*β*, and iNOS. Physiologically, NF-*κ*B remains sequestered in the cytosol by inhibitory kappa B (I*κ*B-*α*) that keeps it inactive. But when I*κ*B-*α* is phosphorylated by a number of different stimuli, NF-*κ*B can be activated to translocate into the nucleus where it interacts with the promoter regions of target genes and enhances their transcription [58]. Previous studies have revealed a contribution of the NF-*κ*B pathway to diabetes-induced retinal inflammation, providing a mechanistic reason to target NF-*κ*B for the treatment of diabetic retinopathy [59]. Consistent with previous studies [11, 27], our results have shown that melatonin reduced the elevated cytoplasmic and nuclear protein expressions of NF-*κ*B (p65 subunit) (Figure 7), associated with the decreased phosphorylation of I*κ*B-*α* (Figure 7). Collectively, these findings demonstrate the anti-inflammatory effect of melatonin in diabetic retina by inhibiting NF-*κ*B. This is consistent with the report about the effect of melatonin on experimental uveitis [60]. It has been revealed that not only are proinflammatory cytokines active downstream in the NF-*κ*B cascade but they also contribute to NF-*κ*B activity [61]. Therefore, besides NF-*κ*B inhibition, melatonin may directly regulate proinflammatory genes via epigenetic on/off mechanisms [62].

A variety of experiments have confirmed that retinal neuron apoptosis is an important component of diabetic retinopathy [63, 64]. The effects of melatonin on RGCs have been reported by many previous studies. Li et al. [65] reported the protection against RGC apoptosis after 8 weeks of diabetes. Others reported that melatonin can promote RGC survival under conditions of retinal ischemia [66]. Therefore, the protective effect of melatonin on the retinal ganglion cells should be analyzed in our further study.

In conclusion, melatonin was found to have potent protective roles in diabetic retinopathy, and the effects were mediated through the attenuation of inflammation by NF-*κ*B inhibition and the prevention of decreased activity of antioxidant enzymes via the Akt-induced Nrf2 pathway. Melatonin also reduced elevated VEGF expression and reversed the retinal dysfunction due to diabetes mellitus. Melatonin appears to have no effect on control nondisease retina [26] and its safety after long-term use has been proved by human clinical study [67]. Thus, melatonin is a candidate compound with potential for therapeutic applications in diabetic retinopathy.

## Figures and Tables

**Table 1:** Primer sequences for RT-PCR analyses.

Genes	Primer	Sequence	Product
TNF-*α*	For	5′-GCCACCACGCTCTTCTGTCTACT-3′	174
	Rev	5′-CGCTTGGTGGTTTGCTACGAC-3′	

IL-1*β*	For	5′-TGGCAACTGTCCCTGAACTCAACTG-3′	251
	Rev	5′-GAAGCTCCACGGGCAAGACATAGGT-3′	

iNOS	For	5′-GCAACATCAGGTCGGCCATTACT-3′	171
	Rev	5′-AGCCCAGGTCGATGCACAAC-3′	

VEGF	For	5′-GTCCAAGATCCGCAGACGTGT-3′	286
	Rev	5′-ACCCAAAGTGCTCCTCGAAGAGT-3′	

*β*-actin	For	5′-CTGAACCCTAAGGCCAACCGTGAAA-3′	274
	Rev	5′-TGAAGCTGTAGCCACGCTCGGTC-3′	

**Table 2:** Effect of melatonin on retinal function. The average amplitudes of scotopic and photopic ERG a-wave and b-wave at 12 wk of treatment were shown. These parameters were significantly reduced in diabetic animals as compared with the control groups, whereas the amplitudes decrease could be inhibited by melatonin treatment. ND, age-matched nondiabetic group; D12, diabetic group at 12 weeks; DM12, melatonin-treated diabetic group at 12 weeks. ^*∗*^ *P* < 0.05 when compared with the ND group; ^#^ *P* < 0.05 when compared with the diabetic group.

Groups	a-wave (uV) (mean ± SD)		b-wave (uV) (mean ± SD)	
	Scotopic ERG	Photopic ERG	Scotopic ERG	Photopic ERG
ND	−122.02 ± 41.29	−37.43 ± 5.54	294.14 ± 28.61	395.83 ± 13.12
D12	−64.52 ± 23.76^*∗*^	−24.21 ± 3.43^*∗*^	193.27 ± 14.95^*∗*^	158.47 ± 19.18^*∗*^
DM12	−121.13 ± 33.08 ^#^	−28.03 ± 3.69	273.48 ± 19.81^#^	277.36 ± 24.39
